# Validation of the Eclipse AAA algorithm at extended SSD

**DOI:** 10.1120/jacmp.v11i3.3213

**Published:** 2010-06-08

**Authors:** Amjad Hussain, Eduardo Villarreal‐Barajas, Derek Brown, Peter Dunscombe

**Affiliations:** ^1^ Department of Medical Physics, Tom Baker Cancer Centre University of Calgary Calgary Alberta Canada T2N 4N2; ^2^ Department of Physics and Astronomy University of Calgary Calgary AB Canada T2N 1N4; ^3^ Department of Radiation Oncology, Tom Baker Cancer Centre University of Calgary Calgary Alberta Canada T2N 4N2

**Keywords:** Eclipse, AAA, extended SSD, TBI

## Abstract

The accuracy of dose calculations at extended SSD is of significant importance in the dosimetric planning of total body irradiation (TBI). In a first step toward the implementation of electronic, multi‐leaf collimator compensation for dose inhomogeneities and surface contour in TBI, we have evaluated the ability of the Eclipse AAA to accurately predict dose distributions in water at extended SSD. For this purpose, we use the Eclipse AAA algorithm, commissioned with machine‐specific beam data for a 6 MV photon beam, at standard SSD (100 cm). The model was then used for dose distribution calculations at extended SSD (179.5 cm). Two sets of measurements were acquired for a 6 MV beam (from a Varian linear accelerator) in a water tank at extended SSD: i) open beam for 5 × 5, 10 × 10, 20 × 20 and $40\times 40\;{\text{cm}}^{2}$ field sizes (defined at 179.5 cm SSD), and ii) identical field sizes but with a 1.3 cm thick acrylic spoiler placed 10 cm above the water surface. Dose profiles were acquired at 5 cm, 10 cm and 20 cm depths. Dose distributions for the two setups were calculated using the AAA algorithm in Eclipse. Confidence limits for comparisons between measured and calculated absolute depth dose curves and normalized dose profiles were determined as suggested by Venselaar et al. The confidence limits were within 2% and 2 mm for both setups. Extended SSD calculations were also performed using Eclipse AAA, commissioned with Varian Golden beam data at standard SSD. No significant difference between the custom commissioned and Golden Eclipse AAA was observed. In conclusion, Eclipse AAA commissioned at standard SSD can be used to accurately predict dose distributions in water at extended SSD for 6 MV open beams.

PACS numbers: 87.53.Kn, 87.55.D‐, 87.55.de, 87.56.ng

## I. INTRODUCTION

Treatment planning for TBI is mainly performed using a point dose calculation approach. In the last decade, however, a number of studies have demonstrated the use of commercially available treatment planning systems for TBI dose distribution calculations.^(^ ^1^ ^–^ ^5^ ^)^ These studies were based on either standard or modified models using data acquired under TBI conditions (i.e. at extended SSD). To the best of our knowledge no such study has been performed with the Eclipse treatment planning system. The Eclipse treatment planning system uses a 3D pencil beam superposition‐convolution algorithm (AAA) for dose calculations.^(^ ^6^ ^,^ ^7^ ^)^ Dose distributions calculated by the AAA algorithm have been thoroughly studied by several investigators for various clinically relevant geometries.^(^ ^8^ ^–^ ^14^ ^)^ For dose distribution calculations in heterogeneous media, and especially at interfaces, the AAA algorithm has been shown to be consistently more accurate than the pencil beam convolution (PBC) algorithm.^(^ ^9^ ^,^ ^10^ ^,^ ^14^ ^–^ ^16^ ^)^

For the commissioning of the Eclipse treatment planning system, the manufacturer advises that the SSD used for treatment planning calculations does not exceed 30% of the SPD (source to phantom distance) used during commissioning of the Eclipse AAA algorithm. If an SSD exceeding the range of 30% is routinely used, it is recommended to acquire another set of measurements at an extended SSD, and to recommission the Eclipse AAA algorithm under these conditions. Based on the limitations of the Eclipse configuration algorithm, the maximum SPD that is allowed for complete beam data storage is 140 cm.^(^ ^17^ ^)^ In the present study, we assess the capability of the Eclipse AAA to accurately predict dose distributions at distances beyond 140 cm in water while using the beam model commissioned at standard SSD (100 cm) with machine specific beam data. All dose calculations presented in this study were performed with the heterogeneity correction turned on and using a 2.5 mm calculation grid size. Measured and calculated depth dose curves and dose profiles were compared using the criteria established by Venselaar et al.^(^ ^18^ ^)^

## II. MATERIALS AND METHODS

### A. Eclipse treatment planning system

For this study the Eclipse (Varian Medical Systems, Palo Alto, CA) AAA photon algorithm version 8.6.15 was used. The algorithm was commissioned using machine‐specific measured beam data at standard SSD (100 cm). This model is hereafter referred to as the AAA Custom model (AAA‐C). It is worth mentioning that AAA‐C was used for dose calculations at extended SSD without any modifications or adjustments to the default model parameters (Table 1). In order to asses the validity of using Varian (Varian Medical Systems, Palo Alto, CA) Golden beam data for the commissioning of the AAA algorithm, calculations were also performed using the AAA algorithm commissioned with Varian Golden beam data. This model is hereafter referred to as the AAA Golden model (AAA‐G). The rationale for addressing the AAA‐G is that the information may be relevant for users of Eclipse‐AAA and Varian Linacs.

**Table 1:** The default parameter values calculated by the AAA configuration algorithm.

*Parameter*	*21EX (6MV)*
Secondary source size	30.4 mm
Secondary source relative intensity	0.026
Mean energy of secondary source	0.73 MeV
$\delta_{o}$ at 100 cm for electron contamination	12.4 mm
$\delta_{1}$ at 100 cm for electron contamination	92.8 mm
Relative weight of $\delta_{o}$ for electron contamination	0.1673

In AAA, the primary photons, scattered photons and electrons scattered from the beam limiting devices are modeled separately. The primary photon source is the point source located at the target plane. These primary photons are the bremsstrahlung X‐rays produced in the target that do not interact in the head of the treatment unit. The primary photon source is represented by an energy spectrum, mean energy radial curve and intensity profile.

The secondary photon source is a virtual plane source located at the bottom of the flattening filter and is modeled by a single Gaussian curve. It models the photons scattered from the flattening filter and collimators. The secondary photon source is characterized by its size, relative intensity and mean energy. The dominant parameter of the secondary source is its relative intensity with respect to the primary source.

The electron contamination source, located at the target plane, models the electrons generated in the treatment unit head and in air (mainly through Compton scattering). This source is characterized by two Gaussians and one depth‐dependent dose deposition curve. The Gaussian functions determine the lateral spread of electrons and the field size dependence of the electron dose distribution. The curve is empirically derived from the difference between the measured depth dose curve and the calculated depth dose curve for the largest field size without electron contamination. A more detailed description of the AAA algorithm is presented elsewhere.^(^ ^6^ ^,^ ^7^ ^,^ ^12^ ^,^ ^19^ ^)^

The configuration algorithm in AAA during the optimization phase, processes the measured data and generates calculated data which is then used as an input for dose calculations. The final dose distribution is the superposition of photon and electron dose convolutions.^(^ ^6^ ^,^ ^20^ ^)^

### B. Experimental measurements

All of the measurements presented were acquired in a 48cm×48cm×41 cm water tank. Two CC13 water proof ionization chambers were used for relative dosimetry measurements. For absolute dosimetry measurements, an Exradin A12 Farmer ionization chamber (Standard Imaging Inc., Middleton, WI) was used following the TG‐51 protocol.^(^ ^20^ ^)^ Two sets of measurements were acquired at extended SSD. The first set of measurements was obtained for open beam field sizes of 5 × 5, 10 × 10, 20 × 20 and $40\times 40\;{\text{cm}}^{2}$. A second measurement set was obtained for the same field sizes but with a 1.3 cm thick acrylic spoiler placed 10 cm above the water surface (distance from the source to the top of spoiler was 168.2 cm). For TBI treatments, the spoiler is used to increase surface dose.^(^ ^21^ ^)^ All measurements were made using 6 MV X‐rays (from a Varian linear accelerator). Percent depth dose curves (PDDs) were measured at extended SSD for both setups. In‐plane and cross‐plane profiles were also acquired for the stated field sizes at 5 cm, 10 cm and 20 cm depths. The field sizes for all extended SSD measurements were defined at 179.5 cm SSD (e.g. for a $10\times 10\;{\text{cm}}^{2}$ field size, the collimator setting was $5.6\;\times \;5.6\;{\text{cm}}^{2}$).

For calculations, a homogeneous water phantom was created in the Eclipse treatment planning system with and without a 1.3 cm thick acrylic spoiler, 10 cm above the water surface. Source to water surface distance was 179.5 cm for both extended SSD setups. Machine output at extended SSD was determined following the TG‐51 protocol and using the relation:^(^ ^20^ ^)^

(1) $$\text{Output}=\frac{\text{Measured}\;\text{Dose}\;\text{per}\;\text{MU}\;(\text{SSD}=179.5\text{cm},\;10\times 10,\;d=10\text{cm})}{\text{Measured}\;\text{Dose}\;\text{per}\;\text{MU}(\text{SSD}=90\text{cm},\;10\times 10,\;d=d_{\text{max}})}\times (1\text{cGy}/\text{MU})$$

The absolute dose per MU measured at extended SSD was divided by the machine output at standard SSD to account for variations in nominal machine output (1cGy/MU). Relative output factors (ROFs) were then determined for other field sizes as a ratio of the ionization current with respect to the reference field size ($10\times 10\;{\text{cm}}^{2}$) at 10 cm depth. These ROFs were used to determine the absolute output at 10 cm depth for other field sizes.

The measured absolute doses at 10 cm depth were used to produce absolute depth dose curves from the measured PDDs. Absolute depth dose curves were calculated for 100 MU in Eclipse AAA and compared with measured absolute depth dose curves. Measured and calculated profiles at 5 cm, 10 cm and 20 cm depths were normalized to 100% at the central axis for relative comparisons.

### C. Data analysis

**Figure 1:**
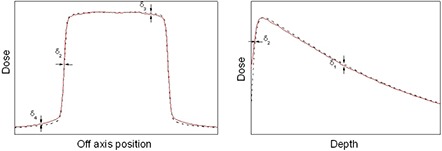
Regions of interest for the determination of confidence limits for depth dose curves and dose profiles.

The analysis was performed over five regions, as shown in Fig. 1. These regions are defined below.

- Build down ($\delta_{1}$): dose deviation on central axis beyond the depth of maximum dose ($d_{\max}$)
- Build‐up and penumbra ($\delta_{2}$): dose deviation on central axis before the depth of $d_{\max}$ (phantom surface to depth of the 90% dose surface) and in penumbra (where dose gradient is larger than 3% per mm)
- Off‐axis ($\delta_{3}$): dose deviation in the inner field at off‐axis points and beyond $d_{\max}$
- Tail ($\delta_{4}$): dose deviation in region outside the geometrical beam edges (less than 7% of the central axis dose)

The percentage difference between measurements and calculations is given by

(2) $$Difference\;(\%)=\frac{\left(D_{calc}-D_{meas}\right)}{D_{meas}}\times 100$$

where $D_{\mathrm{calc}}$ and $D_{\mathrm{meas}}$ are the locally calculated and measured doses (absolute for depth dose curves and relative for profiles).

In region $\delta_{4}$, the deviation was determined with respect to central axis dose as follows:

(3) $$Difference\;(\%)=\frac{\left(D_{calc}-D_{meas}\right)}{D_{meas,central\;axis}}\times 100$$

The confidence limits suggested by Venselaar et al.^(^ ^18^ ^)^ were adopted as tolerance levels when comparing measured and calculated depth dose curves and dose profiles. The confidence limit is defined as:

(4) Δ=|mean deviation|+1.5×SD

where SD is the standard deviation of the differences between calculated and measured data points.

## III. RESULTS & DISCUSSION

**Figure 2:**
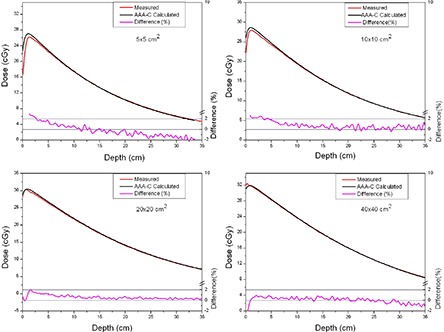
Comparison of absolute depth dose curves for 5 × 5, 10 × 10, 20 × 20 and $40\times 40\;{\text{cm}}^{2}$ field sizes at extended SSD, with the spoiler in the beam. The red line shows measurements and the black line shows curves calculated using AAA‐C. The percentage difference is shown by the magenta line on a scale from −2% to +2% (on right side).

**Figure 3:**
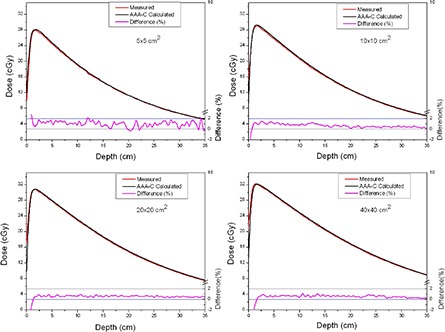
Comparison of absolute depth dose curves for 5 × 5, 10 × 10, 20 × 20 and $40\times 40\;{\text{cm}}^{2}$ field sizes at extended SSD and without the spoiler in the beam.

**Figure 4:**
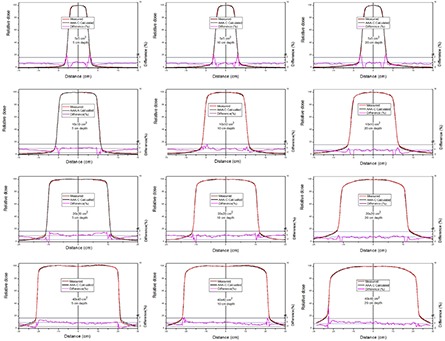
Comparison of normalized dose profiles (at 5, 10 and 20 cm depths) for 5 × 5, 10 × 10, 20 × 20 and $40\times 40\;{\text{cm}}^{2}$ fields at extended SSD, with the spoiler in the beam. The red line shows measurements and the black line shows profiles calculated using AAA‐C. The percentage difference is shown by the magenta line on a scale from −3% to +2% (on right side).

**Figure 5:**
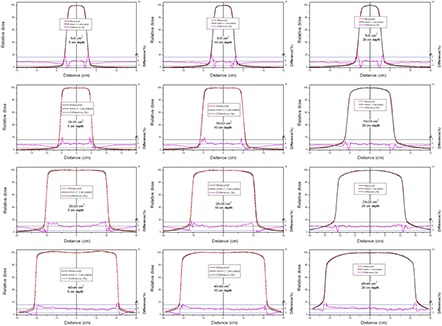
Comparison of normalized dose profiles (at 5, 10 and 20 cm depths) for 5 × 5, 10 × 10, 20 × 20 and $40\times 40\;{\text{cm}}^{2}$ field sizes at extended SSD without the spoiler in the beam.

**Figure 6:**
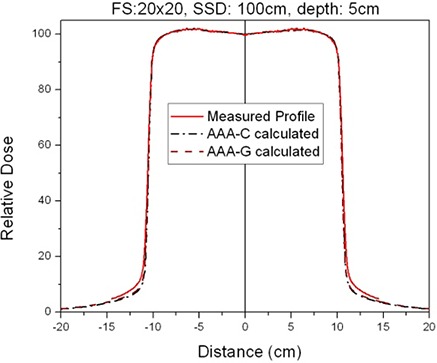
Comparison of measured and calculated profiles at 100 cm SSD and 5 cm depth.

**Figure 7:**
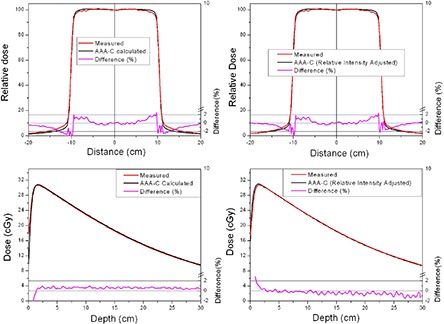
Effect of adjusting the relative intensity of the secondary source in the beam configuration model. The dose profiles and depth dose curves are for a $20\times 20\;{\text{cm}}^{2}$ field size without the spoiler in the beam.

The comparison between measured and calculated absolute depth doses for 5 × 5, 10 × 10, 20×20 and $40\times 40\;{\text{cm}}^{2}$ field sizes at extended SSD are shown in Figs. 2 and 3, with and without the spoiler present, respectively. All depth dose curves are presented in absolute dose, which inherently include MU calculation accuracy. A comparison of calculated and measured profiles (normalized to 100% on central beam axis) at depth of 5 cm, 10 cm and 20 cm with and without the spoiler in the beam is shown in Figs. 4 and 5, respectively. The average percent deviation for the profiles within the field ($\delta_{3}$) was less than 1%. Outside the primary beam in the tail region ($\delta_{4}$), the deviation was less than 2%. It is clear that Eclipse AAA underestimates the dose in the tail region for all the profiles. It should be noted, however, that this underestimation was also observed at standard SSD (Fig. 6). The shape of the tail region and its dose magnitude is characterized by the secondary photon source in AAA model. This systematic underestimation on the tail region confirms the observation by Zhu and Bjärngard^(^ ^22^ ^)^ that a single Gaussian model for the secondary photon source leads to an underestimation of the dose outside the primary beam as showed in Figs. 4 and 5. Furthermore, a model with two Gaussian components has been shown to calculate the scattered photon reasonably well in this region.^(^ ^22^ ^)^ In order to demonstrate how the secondary source parameters affect the tail region of the profile, the value of the relative intensity (the dominant parameter) was changed from the default value of 0.026 to 0.050. The marginal improvement in terms of agreement between calculation and measurements is shown in Fig. 7. However, this change in the relative intensity also affected the depth dose curve, worsening the agreement between calculations and measurements. Therefore, care should be taken while adjusting the model parameters. The effect of the other two parameters (source size and mean energy) of the secondary photon source on both PDDs and dose profiles was negligible.

As shown in Figs. 2 and 3, the AAA‐C slightly overestimates the absolute dose at extended SSD. This overestimation was more dominant (up to 1.8%) for the $5\times 5\;{\text{cm}}^{2}$ field size, but was still within the Venselaar's suggested tolerances.^(^ ^18^ ^)^ Due to the oversimplified modeling of the electron contamination in AAA, deviations in the buildup area were expected. For the setup with spoiler, the acrylic sheet absorbs most of the electrons generated in air, but it also introduces electrons into the beam. These electrons produced in the acrylic increase the surface dose, which is evident in Fig. 2 where $d_{\max}$ is very close to the surface. An overestimation of dose at standard SSD in an inhomogeneous medium at the lung and tissue interface has also been observed by Van Esch et al.^(^ ^12^ ^)^ The deviations between calculated and measured depth doses were higher in this geometry (up to 2.2%) than with the simple open beam (up to 1.8%). The agreement between measurements and calculations was improved as the field size increased.

The confidence limits with their recommended tolerances^(^ ^18^ ^)^ for 5 × 5, 10 × 10, 20 × 20 and $40\times 40\;{\text{cm}}^{2}$ depth dose curves and beam profiles are given in Tables 2, 3, 4 and 5. All of the AAA‐C calculations for depth dose curves and profiles were within the recommended tolerances. AAA‐G predictions were also within tolerance, except for region δ1 for 5 × 5 and $10\times 10\;{\text{cm}}^{2}$ field sizes (with spoiler) where a 2.2% difference was observed. It is worth mentioning that for the depth dose curves with a spoiler in the beam (see Table 2), the confidence limit definition for region δ2 is not applicable because the surface dose is higher than 90%. Both AAA‐C and AAA‐G models were also compared for dose calculation accuracy at standard SSD. A small improvement, in terms of agreement with measured absolute PDDs, was observed in the AAA‐C over the AAA‐G (Table 5). However, in a comparison of the profiles, AAA‐G performed slightly better than AAA‐C.

**Table 2:** Confidence limits for absolute depth dose curves calculated for the AAA ‐C and AAA‐G at extended SSD.

δ*s*	*Field Size cm^2^*	*Open beam, extended SSD*		*Spoiler in the beam, extended SSD*		*Tolerance*
		*AAA‐C* a	*AAA‐G* b	*AAA‐C*	*AAA‐G*	
	5×5	1.4%	1.8%	1.7%	2.2%	2%
$\delta_{1}$	10×10	1.2%	1.7%	1.7%	2.1%	2%
	20×20	0.8%	1.2%	1.3%	1.3%	2%
	40×40	0.9%	1.4%	0.8%	0.8%	2%
	5×5	1.0 mm	1.2 mm	4 mm	4 mm	2 mm
$\delta_{2}$	10×10	1.0 mm	1.0 mm	N/A	N/A	2 mm
	20×20	1.2 mm	1.0 mm	N/A	N/A	2 mm
	40×40	1.2 mm	1.0 mm	N/A	N/A	2 mm

a AAA commissioned with machine specific beam data.

b AAA commissioned with Varian Golden beam data; N/A: Not Applicable.

The confidence limits are presented for the absolute depth dose curves for 5×5, 10 × 10, 20 × 20 and $40\times 40\;{\text{cm}}^{2}$ field sizes at extended SSD in a 6 MV open beam and with the spoiler in the beam.

**Table 3:** Confidence limits for relative dose profiles calculated by the AAA‐C at extended SSD.

δ*s*	*Field Size cm^2^*	*Open beam, extended SSD AAA‐C*		*Spoiler in the beam, extended SSD AAA‐C*		*Tolerance*
		*Depth: 5 cm*	*Depth: 10 cm*	*Depth: 5 cm*	*Depth: 10 cm*	
	5×5	0.3 mm	0.6 mm	0.4 mm	0.3 mm	2 mm
$\delta_{2}$	10 × 10	0.5 mm	0.4 mm	0.3 mm	0.3 mm	2 mm
	20 × 20	0.6 mm	0. 8 mm	0.5 mm	0.6 mm	2 mm
	40 × 40	0.9 mm	0.2 mm	0.2 mm	0.3 mm	2 mm
	5 × 5	0.8%	0.7%	1.5%	1.3%	2%
$\delta_{3}$	10 × 10	0.8%	0.8%	1.6%	1.5%	2%
	20 × 20	1.5%	1.4%	1.1%	1.1%	2%
	40 × 40	0.9%	0.9%	0.9%	0.9%	2%
	5 × 5	1.7%	1.8%	0.7%	0.8%	3%
$\delta_{4}$	10 × 10	2.1%	2.0%	1.8%	1.6%	3%
	20 × 20	2.1%	2.0%	2.0%	1.8%	3%
	40 × 40	1.8%	1.7%	1.9%	1.5%	3%

The confidence limits are presented for the relative profiles at 5 cm and 10 cm depth for 5 × 5, 10 × 10, 20 × 20 and $40\times 40\;{\text{cm}}^{2}$ field sizes at extended SSD in a 6 MV open beam and with the spoiler in the beam.

**Table 4:** Confidence limits for relative dose profiles calculated by the AAA‐G at extended SSD.

δ*s*	*Field Size cm^2^*	*Open beam, extended SSD AAA‐G*		*Spoiler in the beam, extended SSD AAA‐G*		*Tolerance*
		*Depth: 5 cm*	*Depth: 10 cm*	*Depth: 5 cm*	*Depth: 10 cm*	
	5 × 5	0.5 mm	0.6 mm	0.7 mm	0.5 mm	2 mm
$\delta_{2}$	10 × 10	0.6 mm	0.4 mm	0.9 mm	0.4 mm	2 mm
	20 × 20	0.3 mm	0.6 mm	0.6 mm	0.5 mm	2 mm
	40 × 40	0.8 mm	0.4 mm	0.5 mm	0.7 mm	2 mm
$\delta_{3}$	5 × 5	0.8%	0.7%	1.3%	1.5%	2%
	10 × 10	0.9%	0.8%	1.5%	1.6%	2%
	20 × 20	1.4%	1.4%	1.1%	0.7%	2%
	40 × 40	1.2%	0.9%	0.9%	0.9%	2%
	5 × 5	1.7%	1.8%	0.7%	0.8%	3%
$\delta_{4}$	10 × 10	1.9%	2.0%	1.6%	1.5%	3%
	20 × 20	1.9%	1.9%	1.8%	1.2%	3%
	40 × 40	1.5%	1.7%	1.9%	1.3%	3%

The confidence limits are presented for the relative profiles at 5 cm and 10 cm depth for 5 × 5, 10 × 10, 20 × 20 and $40\times 40\;{\text{cm}}^{2}$ field sizes at extended SSD in a 6 MV open beam and with the spoiler in the beam.

**Table 5:** Confidence limits for absolute depth dose curves and relative dose profiles at standard SSD (100 cm).

*Open beam, standard SSD (100 cm)*							
	*Depth Dose Curves*				*Dose Profiles*		
δ*s*	*Field Size cm^2^*	*AAA‐C*	*AAA‐G*	δ*s*	*Field Size cm^2^*	*AAA‐C*	*AAA‐G*
	5 × 5	0.5%	0.7%		5 × 5	0.4 mm	0.6 mm
$\delta_{1}$	10 × 10	0.5%	0.6%	$\delta_{2}$	10 × 10	0.4 mm	0.5 mm
	20 × 20	0.3%	0.6%		20 × 20	0.5 mm	0.6 mm
	40 × 40	0.4%	0.6%		40 × 40	0.6 mm	0.4 mm
	5 × 5	1.0 mm	1.6 mm		5 × 5	1.1%	0.9 mm
$\delta_{2}$	10 × 10	0.8 mm	1.5 mm	$\delta_{3}$	10 × 10	1.1%	1.0%
	20 × 20	0.6 mm	1.5 mm		20 × 20	0.5%	0.4%
	40 × 40	0.8 mm	2.0 mm		40 × 40	0.9%	1.2%
					5 × 5	1.5%	1.4%
				$\delta_{4}$	10 × 10	1.8%	1.6%
					20 × 20	1.7%	1.5%
					40 × 40	1.4%	1.3%

Preliminary dose distribution measurements in an inhomogeneous anthropomorphic phantom (RANDO) (The Phantom Laboratory, Salem, NY) at extended SSD (185 cm SSD) have been performed for comparison with the AAA‐C algorithm described in this study. An opposed beam pair (AP‐PA) was delivered at a zero gantry with a large field ($55\times 42\;{\text{cm}}^{2}$) to cover the head and chest regions. TLDs and radiochromic films were used for dose measurements. The agreement between measurement and calculation was within ±2%, except for the lung region where 3% to 4.9% overestimation was observed. Breitman et al.^(^ ^11^ ^)^ has previously reported a similar dose overestimation of the dose to the chest region in RANDO at standard SSD.

## IV. CONCLUSIONS

The accuracy of the Eclipse AAA algorithm to predict dose distributions in water at extended SSD has been evaluated in preparation for total body irradiation calculations. Confidence limits as suggested by Venselaar et al. were used to evaluate the ability of the Eclipse AAA algorithm^(^ ^18^ ^)^ to accurately predict dose distributions. The results showed that Eclipse AAA, commissioned with standard SSD beam data, accurately predicts dose distributions in water for open, 6 MV X‐ray beams at extended SSD. No significant differences were observed between dose calculations using the AAA model commissioned with Varian Golden data or machine‐specific measured data. This consistency between AAA‐C and AAA‐G dose calculations is not unexpected, given the agreement of the locally measured depth doses and profiles in water and the Varian Golden Data. A preliminary study of the accuracy of the dose distribution predictions from the Eclipse AAA at extended SSD for the heterogeneous anthropomorphic phantom RANDO showed good agreement with TLDs and radiochromic film measurements, except in the chest region where Eclipse AAA overestimated the measured doses by as much as 4.9%.

## References

[1] Abraham D , Colussi V , Shina D , Kinsella T , Sibata C . TBI treatment planning using the ADAC pinnacle treatment planning system. Med Dosim. 2000;25(4):219–24. 11150693 10.1016/s0958-3947(00)00049-2

[2] Chrétien M , Cöte C , Blais R , et al. A variable speed translating couch technique for total body irradiation. Med Phys. 2000;27(5):1127–30. 10841419 10.1118/1.598978

[3] Hui SK , Das RK , Thomadsen B , Henderson D . CT‐based analysis of dose homogeneity in total body irradiation using lateral beam. J Appl Clin Med Phys. 2004;5(4):71–79. 10.1120/jacmp.v5i4.1980 PMC5723515 15738922

[4] Lavallée MC , Gingras L , Chrétien M , Aubin S , Cöté C , Beaulieu L . Commissioning and evaluation of an extended SSD photon model for PINNACLE3: an application to total body irradiation. Med Phys. 2009;36(8):3844–55. 19746817 10.1118/1.3171688

[5] Sánchez‐Nieto B , Sánchez‐Doblado F , and Terrón JA . A CT‐aided PC‐based physical treatment planning of TBI: a method for dose calculation. Radiother Oncol. 1997;42(1):77–85. 9132830 10.1016/s0167-8140(96)01857-9

[6] Ulmer W , Harder D . Applications of a triple Gaussian pencil beam model for photon beam treatment planning. Z Med Phys. 1996;6:68–74.

[7] Sievinen J , Ulmer W , Kaissl W . AAA Photon Dose Calculation Model in Eclipse. Varian RAD# 7170B. Palo Alto, CA: Varian Medical Systems; 2005.

[8] Cozzi L , Nicolini G , Vanetti E , et al. Basic dosimetric verification in water of the anisotropic analytical algorithm for Varian, Elekta and Siemens linacs. Z Med Phys. 2008;18(2):128–35. 18705613 10.1016/j.zemedi.2007.09.003

[9] Bragg CM , Wingate K , Conway J . Clinical implications of the anisotropic analytical algorithm for IMRT treatment planning and verification. Radiother Oncol. 2008;86(2):276–84. 18249453 10.1016/j.radonc.2008.01.011

[10] Rønde HS , Hoffmann L . Validation of Varian's AAA algorithm with focus on lung treatments. Acta Oncol. 2009;48(2):209–15. 18803058 10.1080/02841860802287108

[11] Breitman K , Rathee S , Newcomb C , et al. Experimental validation of the Eclipse AAA algorithm. J Appl Clin Med Phys. 2007;8(2):76–92. 17592457 10.1120/jacmp.v8i2.2350 PMC5722411

[12] Van Eesh A , Tillikainen L , Pyykkonen J , et al. Testing of the analytical anisotropic algorithm for photon dose calculation. Med Phys. 2006;33(11):4130–48. 17153392 10.1118/1.2358333

[13] Fogliata A , Nicolini G , Vanetti E , Clivio A , Cozzi L . Dosimetric validation of the anisotropic analytical algorithm for photon dose calculation: fundamental characterization in water. Phys Med Biol. 2006;51(6):1421–38. 16510953 10.1088/0031-9155/51/6/004

[14] Panettieri V , Barsoum P , Westermark M , Brualla L , Lax I . AAA and PBC calculation accuracy in the surface build‐up region in tangential beam treatments. Phantom and breast case study with the Monte Carlo code PENELOPE. Radiother Oncol. 2009;93(1):94–101. 19541380 10.1016/j.radonc.2009.05.010

[15] Gagné IM , Zavgorodni S . Evaluation of the analytical anisotropic algorithm in an extreme water‐lung interface phantom using Monte Carlo dose calculations. J Appl Clin Med Phys. 2007;8(1):33–46. 10.1120/jacmp.v8i1.2324 PMC5722400 17592451

[16] Bragg CM , Conway J . Dosimetric verification of the anisotropic analytical algorithm for radiotherapy treatment planning. Radiother Oncol. 2006;81(3):315–23. 17125862 10.1016/j.radonc.2006.10.020

[17] Eclipse Algorithms Reference Guide, P/N B500298R01C. Palo Alto, CA: Varian Medical Systems; 2007.

[18] Venselaar J , Welleweerd H , Mijnheer B . Tolerances for the accuracy of photon beam dose calculations of treatment planning systems. Radiother Oncol. 2001;60(2):191–201. 11439214 10.1016/s0167-8140(01)00377-2

[19] Ulmer W , Harder D . A triple gaussian pencil beam model for photon beam treatment planning. Z Med Phys. 2005;5:25–30.

[20] Almond PR , Biggs PJ , Coursey BM , et al. AAPM's TG‐51 protocol for clinical reference dosimetry of high‐energy photon and electron beams. Med Phys. 1999;26(9):1847–70. 10505874 10.1118/1.598691

[21] Gerig LH , Szanto J , Bichay T , Genest P . A translating‐bed technique for total‐body irradiation. Phys Med Biol. 1994;39(1):19–35. 7651996 10.1088/0031-9155/39/1/002

[22] Zhu TC , Bjärngard BE . Head scatter off‐axis for megavoltage x rays. Med Phys. 2003;30(4):533–43. 12722805 10.1118/1.1556609

